# Deep Brain Stimulation: Psychological and Neuroethical Perspectives

**DOI:** 10.3390/neurolint17100158

**Published:** 2025-10-02

**Authors:** Stella Sremic, Antea Krsek, Lara Baticic

**Affiliations:** 1 Department of Clinical, Health and Organizational Psychology, Clinical Hospital Centre Rijeka, 51000 Rijeka, Croatia; stella.sremi@gmail.com; 2 Faculty of Medicine, University of Rijeka, 51000 Rijeka, Croatia; tea.krsek@gmail.com; 3 Department of Medical Chemistry, Biochemistry and Clinical Chemistry, Faculty of Medicine, University of Rijeka, 51000 Rijeka, Croatia

**Keywords:** cognitive functions, deep brain stimulation, emotions, identity, mood, neuroethics, personality, self-perception

## Abstract

Deep brain stimulation (DBS) is an evolving neurosurgical treatment, originally developed for movement disorders such as Parkinson’s disease, essential tremor, and dystonia. In recent years, it has been increasingly applied to psychiatric and cognitive disorders. This review aimed to summarize the psychological and neuroethical dimensions of DBS, with particular attention to cognitive, emotional, and personality-related outcomes. While DBS can significantly enhance quality of life, it may also lead to subtle or overt changes in cognition, affect, and self-perception, especially in patients with neuropsychiatric comorbidities. Comprehensive psychological evaluation, both pre- and post-operatively, is essential. Findings from recent trials highlight a balance of potential risks and benefits that must be communicated transparently to patients. From a neuroethical perspective, DBS raises important questions regarding personal identity and autonomy, concerns that will become increasingly relevant as the technology advances. This paper underscores the need for more systematic research and the development of personalized care protocols that address not only motor outcomes but also psychosocial well-being.

## 1. Deep Brain Stimulation

**Figure 1:**
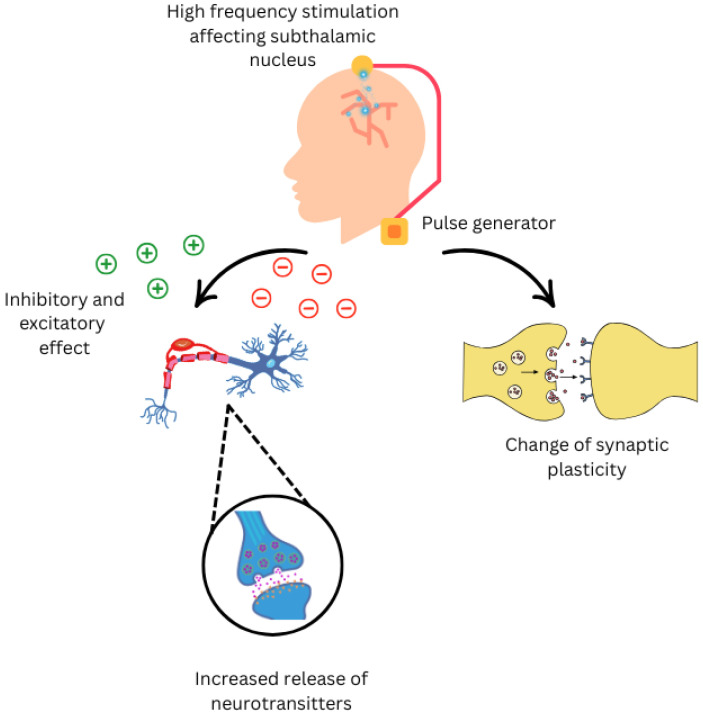
Neurological effects of Deep Brain Stimulation on nervous system.

Deep brain stimulation (DBS) is a neurosurgical technique that has been in clinical use for several decades and continues to undergo significant refinement. It is well established as an effective therapy for neurological disorders such as Parkinson’s disease (PD) [1], essential tremor (ET) [2], and dystonia [3]. The U.S. Food and Drug Administration (FDA) first approved DBS in 1997 for the treatment of ET, followed by approvals for PD in 2002 and for dystonia in 2003, the latter under a Humanitarian Device Exemption (HDE) [4]. Beyond its efficacy in movement disorders, DBS has also been investigated and applied for symptom relief in conditions such as epilepsy, chronic pain, Tourette syndrome, and several psychiatric disorders [4,5,6]. DBS implies a surgical procedure in which a pulse generator is permanently implanted, typically below the clavicle, from which electrical impulses are transmitted to electrode(s) in the brain [4,7,8]. The appropriate target location for electrode placement is a disease-relevant brain region which depends on multiple factors such as the patient’s symptoms, age and cognitive status [7,9]. Several models and hypotheses have been proposed to explain the mechanisms underlying DBS [6,10,11,12]. A central tenet across these perspectives is that electrical stimulation restores function within disrupted neural circuits [5,11]. However, the effects of DBS are highly complex and multifactorial, encompassing immediate neuromodulatory influences, activity-dependent synaptic plasticity, and long-term neuronal reorganization [6,11,13]. In recognition of this broader scope, some authors have even suggested a shift in terminology—from deep brain stimulation to deep brain neuromodulation (DBN) [6]. Nonetheless, DBS’s reversible, non-ablative and adjustable nature makes it a preferable alternative to lesion therapy [5,10]. It is important to note that the procedure is not completely risk-free nor without potential adverse effects despite its advantages (Figure 1). Some of the possible complications include infection, hemorrhage, hardware-related complications, cognitive and psychiatric symptoms, motor dysfunction and morbidity [5,7,8,14,15,16]. In order to reduce the risk of potential side effects, thorough patient selection guided by a multidisciplinary team is required [17,18,19,20,21]. Post-surgically, patients undergoing DBS may experience changes in cognitive functioning [22], mood and emotional regulation [23], as well as in aspects of personality and self-perception [24].

Current perspectives on DBS are rapidly evolving beyond the traditional “functional lesion” model, where stimulation was thought to simply inhibit pathological neural activity. New theories propose that DBS acts as a neuromodulatory informatic intervention, capable of disrupting pathological network oscillations and restoring more natural, dynamic neural communication across entire brain circuits, such as the cortico-striato-thalamo-cortical loop. This shift towards a circuit-based understanding is driving a more personalized approach. Instead of continuous stimulation at a fixed target, closed-loop or adaptive DBS systems are being developed. The risk of cognitive and neuropsychiatric side effects following DBS is multifactorial. Susceptibility is influenced by a combination of patient-related factors, disease-related factors and stimulation-related factors. To identify at-risk individuals, a comprehensive baseline neuropsychological and psychiatric evaluation is critical for preoperative selection. Postoperatively, proactive, long-term follow-up is essential to monitor for adverse effects, allowing for timely intervention through stimulation adjustment and supportive therapies to optimize outcomes and mitigate risk [20,21,22,23,24]. This article aimed to explore the aforementioned psychological changes associated with DBS while emphasizing the importance of neuroethics and the general ethical narrative surrounding its application.

To better synthesize the heterogeneous findings on psychological outcomes of DBS, we summarized evidence across disorders, stimulation targets, and follow-up durations (Table 1). The table outlines reported effects on cognitive domains, mood and emotional regulation, and personality- or identity-related changes, alongside factors influencing neuropsychiatric outcomes and strategies for clinical monitoring.

## 2. Cognitive Outcomes Following DBS

Identifying suitable candidates for deep brain stimulation (DBS) requires a multidisciplinary team approach, incorporating comprehensive clinical and neuropsychological assessments [17,21,58]. One of the key selection criteria is the patient’s preoperative cognitive functioning [21,39,59]. Evidence suggests a positive correlation between higher baseline cognitive performance and greater motor improvement in PD patients undergoing bilateral subthalamic nucleus (STN) DBS [59]. Conversely, significant cognitive impairment or established dementia are widely regarded as contraindications for DBS [60,61], as the surgical risks in such cases are likely to outweigh potential therapeutic benefits, potentially offering greater efficacy, fewer side effects, and insights into the fundamental neurophysiology of neurological and psychiatric disorders [58]. This is primarily attributed to potential difficulties in the safe electrode implementation due to cerebral atrophy, concern that those patients could be at greater risk of post-surgical accelerated cognitive deterioration, post-surgical confusion, prolonged hospitalization and being admitted to a nursing home. Moreover, due to cognitive decline, the patient may not be adequately able to participate in the DBS procedure itself, which could consequently lead to inaccurate electrode placement and suboptimal device programming [60]. On the other hand, even though lower baseline cognition is connected to worse DBS outcomes [22], according to expert consensus in selected cases the surgery could be offered if deemed appropriate (for example, for pal-liative care) [60,61].

While most research to date has been directed toward optimizing motor functions, the evolution of DBS techniques has led to a more detailed investigation of its other impacts, including cognitive. This aspect warrants attention, given that even subtle declines in cognitive function can significantly reduce patients’ quality of life [36]. The literature on cognitive outcomes after DBS reports varying results depending on the targeted brain region and methodologies [22,36]. Therefore, some meta-analyses showed a small, yet statistically significant decline in global cognition, whereas other studies reported no changes in global cognition following DBS [22]. Nonetheless, evidence consistently in-dicates that STN-DBS is associated with greater cognitive decline compared to globus pallidus internus (GPi) DBS [22,36,37]. The latter may be attributed to the smaller size of STN and its complex anatomical organization, increasing the likelihood of unintended stimulation of non-motor areas [37].

When examining specific cognitive domains, reduced verbal fluency is the most commonly reported in PD patients following DBS [22,36,59], although it has also been noted in cases of ET [36,38]. Certain studies with PD patients indicate impairments in executive functioning [36], while others are inconclusive [22]. Outcomes related to memory in PD patients are similarly heterogeneous, with some studies suggesting de-clines in verbal, nonverbal and visuospatial memory [22,36]. Evidence from the PD re-search indicates declines in attention and processing speed [36], while others yield in-conclusive results [22]. Processing speed may be impaired in dystonia after DBS [36]. Additionally, some studies report a decline in visuospatial functions [36], while others observe no change or even slight improvements [22]. In DBS for other disorders, such as obsessive–compulsive disorder (OCD), studies demonstrate variable outcomes in at-tention, memory, executive functions and cognitive flexibility, but the procedure is overall regarded as cognitively safe [39]. In patients with treatment-resistant depression (TRD) no cognitive decline was found up to eighteen months of DBS. Some findings even point to slight cognitive improvements in verbal and visual memory, attention, psychomotor speed and executive functioning [40]. Certain studies suggest that DBS may slow cognitive deterioration in individuals with Alzheimer’s disease [51]. Although research on cognitive outcomes involving patients with epilepsy who underwent DBS is limited and remain inconsistent, some results suggest improvements in verbal memory in those with reduced seizure activity [52].

**Figure 2:**
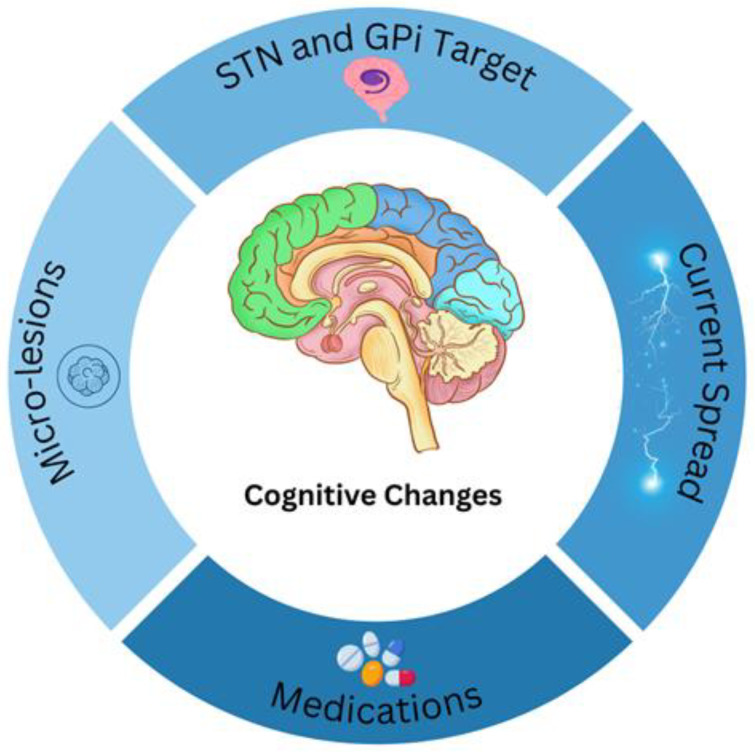
Cognitive changes may arise from numerous factors, and several possible contributors are illustrated here. These include: alterations in medication dosage, unintended current spread to adjacent areas (e.g., GPi stimulation affecting the internal capsule), the specific brain target used for therapy, and micro-lesion effects.

A range of factors must be taken into account when evaluating cognitive functions after DBS (Figure 2). Cognitive outcomes can be influenced by electrode placement and current spread, particularly with STN stimulation, where a more medially placed electrode within the STN has been associated with cognitive decline [37]. Some cognitive changes can stem from microlesions related to electrode placement, while others are induced by the stimulation itself [36]. Furthermore, it is difficult to attribute postoperative decline solely to DBS in cases of a progressive disease such as PD [37]. Patient’s age is an important indicator since older age is a risk factor for postoperative cognitive decline [37,40]. A decrease in medication following DBS may also influence cognitive outcomes [36]. Given the lack of research and the considerable heterogeneity of the limited available data, it is recommended to implement detailed routine assessment of cognitive outcomes following DBS in clinical practice to gain new and important insights in this field and ultimately improve patients’ quality of life.

Cognitive effects are most consistently observed in Parkinson’s disease, particularly declines in verbal fluency following STN stimulation, whereas GPi-DBS appears comparatively safer in this domain. In essential tremor and dystonia, cognitive changes are less pronounced, though subtle deficits in processing speed and fluency have been noted. In psychiatric indications such as OCD and TRD, cognitive functioning is largely preserved, with some studies reporting mild improvements in attention and memory. Mood outcomes display a more complex pattern: while DBS often reduces depression and anxiety symptoms across indications, stimulation of specific sites (e.g., antero-ventral STN) can precipitate transient hypomania, apathy, or irritability. Personality and identity-related effects remain less well understood, though some patients report restoration of a “pre-illness” sense of self, while others experience impulsivity or altered self-esteem, often more evident to caregivers than to patients themselves.

Follow-up duration varies widely across studies, ranging from short-term pilot trials in anorexia nervosa and addiction to multi-year evaluations in PD, OCD, and epilepsy. Importantly, neuropsychiatric outcomes are shaped not only by the stimulation target and parameters but also by patient- and disease-related factors such as age, baseline cognition, psychiatric history, disease progression, and medication adjustments. This highlights the need for standardized pre- and postoperative neuropsychological and psychiatric assessments, alongside long-term monitoring to optimize outcomes and ensure patient safety (Table 1).

## 3. Effects on Mood and Emotions

The anatomical specificity of DBS, wherein electrodes are precisely implanted within circuits governing affect and cognition, necessitates a comprehensive neuropsychiatric evaluation that extends beyond motor or primary symptom assessment. It is crucial to consider that the therapeutic modulation of nodes within networks such as the limbic system or cortico-striato-thalamo-cortical loops can exert profound and sometimes unintended effects on emotional functioning. These effects are not merely side effects but are often intrinsic to the mechanism of action, as DBS fundamentally alters the information processing of neural circuits responsible for mood regulation, motivation, and emotional valence [36,37,38,40,51,52]. Consequently, rigorous pre-operative baseline assessments of emotional and mood states are indispensable prerequisites for patient selection and surgical approval, serving to establish a reference point and contra-indicate individuals at high risk for psychiatric adverse events. Furthermore, post-operatively, continuous monitoring is critical, as the chronic electrical stimulation itself can induce dynamic changes (both beneficial and detrimental) to these same emotional domains. This holistic approach to evaluation ensures not only the safety and efficacy of the procedure but also provides vital insights into the intricate functional organization of human emotional circuitry [25,30,44].

Neuropsychological assessment includes a clinical interview and standardized screening instruments designed to detect any presurgical psychological, emotional or behavioral difficulties [17,44]. Individuals presenting with psychosis or severe active psychiatric conditions are usually excluded [62,63]. Furthermore, severe or untreated depression and/or anxiety are commonly considered a contraindication for the surgery [25,44,62,63]. The practice of screening patients for depression was prompted by evidence from pallidotomy studies, which indicated that a prior history of depression was associated with an elevated risk of postoperative worsening of psychiatric symptoms [63]. In addition, a prior history of bipolar disorder or suicidal ideation and attempts requires particular caution. There were reported suicide cases in patients with bipolar affective disorder or depression following DBS [26,62]. Moreover, the assessment of specific symptoms such as suicidal ideation, anger and panic attacks is also recommended [44].

The effects of DBS on mood and emotional functioning are multifaceted and can differ depending on the stimulation target, the nature of the underlying disorder, and individual patient characteristics. Nevertheless, there is an evident lack of empirical data when it comes to measuring psychological outcomes and associated potential changes. Some patients report a positive experience undergoing DBS, including improved general well-being, mood, self-confidence, hope, and sense of purpose. However, improved mood is frequently related to symptom reduction in the primary disorder. One study showed that negative changes were fewer and transient, while sometimes they could be attributed to the progression of the disease, not the stimulation itself [30]. DBS, in general, leads to a decrease in symptoms of depression in patients [23,25,27,63]. This improvement may be more prominent in patients with a longer duration and more severe initial symptoms [63]. A moderate reduction in negative affect has also been observed [23]. Furthermore, anxiety symptoms are improving [23,25]. The latter could be a consequence of the alleviation of motor symptoms or the effect of stimulation on limbic circuits [23]. Stimulation of the ventral STN has been specifically associated with improvements in anxiety symptoms [28]. On the other hand, apathy symptoms may increase following STN stimulation [23,25,26]. Considering lowering depression, it may appear paradoxical, but it could be pointing out to reduction in filter sensitivity to incoming affectively laden stimuli [23]. Some reports documented acute increases in anger following STN DBS, [27] whereas one study noted a higher incidence of anger or bitterness in the STN DBS group compared to the GPi group [29]. Mania or hypomania may occur, often transiently, particularly in the acute postoperative period, and are associated with stimulation parameters or preexisting psychiatric history [23,27]. If it occurs, it is most often associated with antero-ventral STN stimulation and usually can be managed by adjusting stimulation parameters [27]. Findings regarding facial emotional recognition are inconclusive. Some studies indicate impairments, particularly in the processing of negative emotions, while others report no significant changes [27,64]. Although certain research has not identified an elevated risk, some have reported increased incidence of suicidal ideation and attempts following STN DBS in PD patients [23,25,26].

DBS for psychiatric disorders is still within the scope of clinical trials [41]. The main focus for developing effective closed-loop DBS systems in psychiatry implies the identification of relevant neural signatures and biomarkers associated with mood fluctuations [57]. Among psychiatric disorders, obsessive–compulsive disorder (OCD) is currently the only FDA approved indication (under HDE) for DBS [42]. DBS has been shown to be an effective alternative treatment for severe, treatment-resistant OCD, leading to symptom reduction and improved quality of life [41,43]. At present, a lot of attention is being directed toward ongoing research of DBS efficacy in treating depression, particularly treatment-resistant one in patients who did not respond to standard treatment [45,46,47,48]. Stimulation of the subcallosal cingulate gyrus, nucleus accumbens and medial forebrain bundle has shown promising antidepressant effects, with the medial forebrain bundle yielding the most consistent response [46]. DBS may modulate dysfunctional neural circuits, affect the release and activity of neurotransmitters (serotonin, dopamine, noradrenaline), induce neuroplastic changes and promote neurogenesis and therefore improve symptoms of depression [47]. However, the findings are heterogeneous, as some studies have failed to demonstrate a reduction in depressive symptoms [41,42,46]. The limited number of studies conducted with patients with bipolar disorder report significant improvement in depressive symptoms, although hypomanic symptoms may occur and can be managed through adjustment of stimulation parameters [50]. Furthermore, DBS in patients with anorexia nervosa has shown improvements in BMI, mood, anxiety, affect regulation and associated obsessive-compulsive symptoms in preliminary studies [41,42,54]. Promising preliminary results have also emerged in studies investigating DBS in heroin and cocaine addiction [41,42]. DBS is also being investigated for intrarutine aggressive and self-injurious behaviors, particularly when comorbid with intellectual disability or autism spectrum disorder which has so far shown promising results [55].

Emotional functioning is a crucial determinant of quality of life and as such, warrants more comprehensive investigation. Currently, the lack of evidence is inconclusive, with some studies reporting inconsistent findings. Therefore, it is important to implement thorough pre- and post- operative assessments of mood and emotional functioning across multiple research designs. Furthermore, some authors emphasize that mild to moderate mood symptoms should not necessarily preclude patients from consideration for DBS, as their outcomes have been comparable to those of asymptomatic peers [44].

Table 2 summarizes cognitive outcomes following DBS across neurological and psychiatric disorders. The table contrasts therapeutic and adverse effects, taking into account disorder-related baseline impairments, to help differentiate stimulation-related changes from illness effects. Therapeutic and adverse cognitive effects were reported across major DBS targets and indications. While PD and other neurological disorders most consistently show subtle postoperative declines (particularly in verbal fluency with STN stimulation), psychiatric indications such as OCD and TRD generally demonstrate cognitive safety, with occasional improvements in memory, attention, or executive function. Importantly, these improvements often parallel symptomatic relief rather than direct neuromodulatory effects. In Tourette’s syndrome, cognitive changes remain difficult to isolate due to high rates of psychiatric comorbidity. This comparative perspective underscores the necessity of baseline-adjusted and controlled designs in future research, as well as the routine use of standardized neuropsychological assessments to guide patient selection, optimize stimulation parameters, and interpret long-term outcomes (Table 2).

## 4. Impacts on Personality, Identity and Self-Perception

In addition to the previously mentioned aspects, it is also important to consider the potential ways in which DBS may influence personality, identity and self-perception. There are numerous definitions of personality and various theoretical models that focus on different approaches to studying it. To this day, there is no consensus among scholars, and therefore, no single, universally accepted definition of personality exists [56]. The definition followed in the DSM-5, which concisely explains this construct for the purposes of this paper, describes personality as a persistent pattern of inner experience and behavior [65]. Similarly, over the past few decades, various authors have developed theories to explain the complex construct of identity [66,67]. It can be viewed as a specific form of social representation that mediates the relationship between the individual and the social environment. It consists of three components—self-knowledge, self-directed action and actions of others—through which a person constructs an image of oneself and the world, thereby shaping one’s identity [66]. Lastly, self-perception can be defined as an individual’s experience and evaluation of themselves. Self-respect, a stable self-image and reflective functioning further characterize this construct [67].

Potential DBS candidates often express a degree of concern that the implantation of electrodes could result in unwanted alterations to their personality or sense of self [67]. However, some authors argue that claims regarding personality changes following DBS are more a result of theoretical assumptions rather than empirical evidence and thus lack sufficient evidence [53]. Nonetheless, among the available studies, some suggest that DBS does not significantly alter personality, identity or the subjective experience of self [67]. However, in one study, PD patients exhibited increased levels of depressive symptoms, low self-esteem and work-related difficulties twelve months after undergoing DBS [31]. On the other hand, in patients with TRD, subcallosal cingulate DBS has been associated with decreased neuroticism and increased extraversion [32]. Furthermore, higher preoperative novelty seeking and cooperativeness scores were correlated with improved quality of life one year after STN-DBS in PD patients [33]. One study found that 22% of patients and 44% of caregivers subjectively perceived changes in mood and personality following STN-DBS, even though these changes were not always adequately captured by standardized rating scales [34]. Some reports also indicate personality changes, particularly increased impulsivity, which were primarily observed by family members rather than by the patients themselves [35]. In another study, only a minority of patients with various neurological and psychiatric disorders reported negative experiences, which were mostly temporary or attributed to the disease progression. They dominantly noted positive personality-related changes, including a return to pre-illness state, improved interpersonal relationships and increased self-confidence [30].

More recent qualitative studies suggest that DBS may contribute to the restoration of a sense of identity that the illness had compromised [30,49,68]. Some patients expressed that they felt “more like their former selves,” followed by a greater sense of self-understanding [30,68]. Both participants and their family members emphasized that DBS had helped restore aspects of the identity that had been diminished as a result of the disease [68]. One study indicated that, in the context of PD—which generally has a negative impact on patients’ personality and self-concept—DBS resulted in fewer mostly positive changes, occasionally restoring what was described as the “true self” [24]. TDR patients reported that severe depression had suppressed essential traits of their personality and their sense of self, while DBS provided a sense of hope for returning their previous identity [49].

However, as with previously discussed phenomes such as cognitive functions, emotions and mood, it is difficult to attribute observed changes in personality and identity to a single, specific cause. Besides DBS alone, it is important to consider other contributing factors to these changes, including the medication modifications, progression of the underlying disease, psychosocial adjustment and preoperative personality traits [69,70]. In this context, it is also important to mention the phenomenon known as the “burden of normality,” which may emerge in some patients following DBS [49,71]. PD patients often experience substantial improvements in motor symptoms and objective functioning, yet they may struggle to adapt to a “normal” state, transitioning abruptly from being chronically ill to feeling well, and may find it difficult to cope with the absence of symptoms [71]. The authors emphasize that the latter is not a direct consequence of potential personality changes, but rather that the difficulties in social reintegration after DBS are related to the challenges of patients’ re-establishing themselves within social, familial and work environments [72].

Nevertheless, the scholars emphasize that the literature on personality and identity changes following DBS is very limited and that there is a lack of primary empirical studies supporting the potential effects of DBS in this context [53,67].

## 5. DBS and the Ethical Imperative: Importance of Neuroethics

Neuroethics is an interdisciplinary field that addresses the ethical, philosophical and social implications of neuroscience and neurotechnology. It explores how our growing understanding of the brain and the ability to intervene in its functioning can help us better understand and address complex questions such as those related to personality and identity [73,74]. In doing so, it calls for a stronger empirical foundation and more cautious conclusions in discussions, highlighting those theories should be aligned with existing evidence [53]. As explained in this paper, DBS as a treatment method does not solely cause changes in physical symptoms, but may also have an impact on patients’ cognitive functions, emotions and mood, personality and identity. Neuroethics helps assess whether these changes are a direct consequence of DBS, whereas even if indirect, they still hold important clinical significance and ethical considerations [53,73].

In this context it is essential to highlight Beauchamp and Childress’ principles which are the most widely used framework in biomedical ethics, often referred to as “principlism” [75]. The four fundamental ethical principles outlined are respect for autonomy, non-maleficence, beneficence, and justice [76]. Autonomy can be defined as a higher level of identification with one’s first-order desires, if this identification is not primarily the result of alienating influences [77]. Values are considered inauthentic if they are imposed in situations where individuals are deprived of reasonable alternatives or when such options are morally illegitimate or prohibited. This implies that healthcare professionals should be able to question the authenticity of a patient’s values and preferences, exploring whether the individual can challenge their own values and whether reasonable alternatives were denied to them [77]. New DBS systems, such as volitional closed-loop DBS (VCL-DBS), can give patients active control over stimulation [78]. When the device detects undesirable neural activity, it provides the patient with a visual or auditory signal based on which the patient can decide whether the stimulation will start, increase or be declined [78]. While this may appear to enhance autonomy, it has also raised criticism. The aforementioned issue can further be illustrated by the case of a Dutch patient with PD who experienced euphoria and manic episodes following DBS [79]. If he were equipped with a VCL-DBS system, he might feel an irresistible urge to increase the stimulation to achieve pleasurable mental state, potentially leading him into a self-destructive cycle and risky behaviors [80]. This raises the discussion within the field between balancing the respect of patients’ local autonomy, which refers to the autonomy of an individual decision at a given moment, and global autonomy which is the ability to autonomously pursue goals over a longer period [80]. In such cases, healthcare professionals should carefully assess when the patient is truly capable of making autonomous decisions, considering both the symptoms of the disease and the potential side effects of treatment. For example, clinical depression in general significantly impairs autonomy. Although many patients with depression can make rational decisions, symptoms such as anhedonia and low energy diminish their ability to act according to their will, especially when personal effort is required [81]. However, some authors argue that the DBS itself has the potential to restore autonomy in depressive patients [81]. Therefore, further empirical research is needed, along with an individualized approach to patients, supported by thorough information sharing and effective communication.

Unlike some other neurosurgical procedures, DBS is potentially reversible, which is considered one of its clinical and ethical advantages [82]. This means that patients must actively make decisions about continuing treatment over time, which implies multiple points for obtaining informed consent and assessing decision-making capacity [74]. Throughout this paper, the importance of a multidisciplinary approach has been pointed out, especially due to the potential non-physical changes following the procedure. What is crucial in this approach, as well as in all other areas of patient care, is effective doctor-patient communication. Patients and their caregivers often hold very high and sometimes unrealistic expectations of DBS, viewing it as a “last hope” and “cure-all” solution [83]. Despite improvements in motor symptoms in PD patients, one study found that most caregivers were disappointed with the STN-DBS outcomes, thereby emphasizing the significant impact of unexpected difficulties with fluctuating symptoms and side effects on overall satisfaction [83,84]. Hence, it is an important responsibility of experienced multidisciplinary teams to clearly set expectations for potential DBS undergoing patients [84]. The results of numerous studies indicate that patients with varying conditions are not adequately informed about the different aspects of their illness that affect their quality of life [85]. The consent process should include a detailed discussion of the long-term consequences of DBS and clearly outline the conditions under which it would be recommended to discontinue stimulation or remove the device. Attention should also be given to correcting misconceptions and unrealistic therapeutic expectations [82]. In this process, it is essential to assess how well patients understand the treatment framework, as research has shown that physicians often assume a level of understanding that does not reflect the patient’s actual comprehension, which can lead to significant discrepancies in the communication [85].

Furthermore, with the advancement of DBS technology, particularly the introduction of wireless connectivity and integration with commercial platforms, a new risk has emerged, known as “brainjacking”. The latter phenomenon entails the control of neural activity without the patient’s consent, theoretically via wireless technology. Potential risks include patients’ data misuse, direct interference with the treatment and philosophical, legal and economic implications. Given the rapid advancement of neurotechnology, authors emphasize the importance of initiating neurosecurity discussions, investigating stronger safety measures and educating both clinicians and patients about these risks [8].

## 6. Conclusions

To conclude, the changes following DBS are complex and multifaceted, extending beyond physical symptoms and affecting the psychological and social well-being of patients and their families. The authors highlight the lack of empirical research examining potential cognitive and emotional outcomes, as well as changes in personality and identity, which leads to a limited understanding of these issues. Therefore, it would be beneficial for clinical practice to encourage the development of research designs that focus more closely on the aforementioned processes, which significantly impact patients’ quality of life. Additionally, it would be beneficial to implement pre-DBS educational programs that take into account patients’ personality traits and help them better manage expectations and adapt to life after stimulation. Following this, neuroethics is essential for the responsible conduct of DBS research and treatment, particularly in the context of ethical dilemmas posed by its impact on mental health, as well as emerging security concerns such as “brainjacking”. Detailed and transparent communication with patients, assessment of their understanding and the setting of realistic expectations are crucial for keeping patients’ wellbeing and autonomy.

## Figures and Tables

**Table 1:** Cognitive, Mood, and Personality Outcomes of DBS by Disorder, Target, and Follow-up.

Disorder	Target	Cognitive Outcomes	Mood/Emotional Outcomes	Personality/Identity Outcomes	Follow-up/Notes	Monitoring Factors
Parkinson’s disease (PD)	STN, GPi	Verbal fluency decline most frequent; executive and memory impairments inconsistent; STN > GPi decline [22,25-27,30,31]	↓ Depression, ↓ anxiety, ↑ apathy; transient mania/hypomania (antero-ventral STN); occasional anger outbursts [23,25,26,27,28,29]	Mixed: ↑ depressive symptoms, low self-esteem, impulsivity (often reported by caregivers); some restoration of “true self” [24,30,31,32,33,34,35]	Effects evident up to 12–18 months, some persistent long-term	Age, baseline cognition, electrode placement, medication changes; requires neuropsychological follow-up [20,21,22,23,24,36,37]
Essential tremor (ET)	VIM thalamus	Occasional verbal fluency decline; other domains largely unaffected [36,38]	Limited evidence; some mood improvement via tremor relief	Not systematically studied	Few long-term data	Similar to PD; less pronounced cognitive/mood changes [36]
Dystonia	GPi	Possible decline in processing speed [36]	Mood improvement secondary to motor benefit; apathy risk [36]	Not well studied	Variable	Stimulation spread, disease progression influence outcomes [36]
Obsessive–compulsive disorder (OCD)	Ventral capsule/striatum, NAcc, STN	Variable across domains; overall cognitively safe [36,37,38,39,40]	↓ OCD symptoms, ↓ anxiety, ↑ quality of life [41,42,43]	Some reports of identity restoration [30]	Long-term, FDA-approved under HDE	Continuous psychiatric monitoring; mood effects linked to limbic circuitry [25,30,44]
Treatment-resistant depression (TRD)	Subcallosal cingulate, medial forebrain bundle, NAcc	No decline up to 18 months; mild improvement in memory, attention, psychomotor speed [40]	Antidepressant effects, especially medial forebrain bundle; variable results [45,46,47,48]	↓ Neuroticism, ↑ extraversion; restoration of sense of self [32,49]	12–18 months and longer	Close monitoring of suicidality; parameter adjustments reduce hypomania [50]
Alzheimer’s disease (AD)	Fornix, NBM	Possible slowing of decline; heterogeneous findings [51]	Limited evidence	Identity/self-perception not yet studied	Requires longitudinal follow-up	Stimulation may slow deterioration; evidence scarce [51]
Epilepsy	ANT, hippocampus, CM thalamus	Mixed; some verbal memory improvement with seizure reduction [52]	Mood/affect may improve with seizure reduction [52]	Sparse data	Multi-year studies [53]	Seizure outcome correlates with cognitive/mood benefits [20,52,53]
Anorexia nervosa	Subcallosal cingulate	↑ BMI; improved cognitive control of affect [54,55,56]	↓ Anxiety, improved affect regulation, ↓ OCD symptoms [54,55,56]	Early evidence of improved self-perception [44]	Short-term pilot studies	Monitor for relapse, psychiatric comorbidity [44,54,55,56]
Addiction (heroin, cocaine)	NAcc	Cognitive safety preliminary [41,42]	↓ Craving, improved mood [41,42]	Not studied	Early-phase trials	Need biomarkers for closed-loop DBS [57]

Abbreviations: STN = subthalamic nucleus; GPi = globus pallidus internus; VIM = ventral intermediate nucleus of thalamus; NAcc = nucleus accumbens; ANT = anterior nucleus of thalamus; CM = centromedian nucleus; NBM = nucleus basalis of Meynert.

**Table 2:** Cognitive outcomes following DBS across neurological and psychiatric disorders, with therapeutic vs. adverse effects, compared against disorder-related baseline impairments.

Disorder	DBS Target	Baseline Cognitive Burden	Therapeutic Effects	Adverse Effects	Notes/Differentiating DBS vs. Illness Effects
Parkinson’s disease (PD)	STN, GPi	Executive dysfunction, memory decline, attentional deficits with disease progression [22,36]	Motor improvement can indirectly support cognition via reduced medication load [36]	Decline in verbal fluency (most consistent), possible memory and executive deficits; STN > GPi [22,36,37,59]	Longitudinal designs suggest part of decline reflects disease progression rather than stimulation [37]
Essential tremor (ET)	VIM thalamus	Minimal baseline cognitive impairment [36]	Tremor relief may improve functional capacity [36]	Subtle verbal fluency decline in some patients [38]	Effects generally mild; matched controls show minimal cognitive risk [36,38]
Dystonia	GPi	Generally preserved cognition [36]	Motor symptom relief; indirect QoL benefits [36]	Possible slowing in processing speed [36]	Distinguishing DBS vs. disease effect is difficult due to heterogeneity [36]
Epilepsy	ANT, hippocampus, CM thalamus	Memory and attention deficits common [52]	Verbal memory improvement in seizure responders [52]	Mixed evidence; non-responders may show no benefit [52,53]	Improvement correlates with seizure reduction rather than stimulation [52,53]
Alzheimer’s disease (AD)	Fornix, NBM	Progressive memory and executive decline [51]	Possible slowing of cognitive deterioration [51]	Outcomes inconsistent; some non-responders deteriorate [51]	Cognitive course difficult to dissociate from disease trajectory [51]
Obsessive–compulsive disorder (OCD)	Ventral capsule/striatum, NAcc, STN	Executive dysfunction, cognitive inflexibility, memory deficits [36,39]	Reduction in obsessions improves cognitive efficiency [36,39]	Variable subtle effects on attention and memory; overall cognitively safe [36,37,38,40]	Studies with matched controls suggest most changes reflect illness burden rather than DBS [36]
Treatment-resistant depression (TRD)	Subcallosal cingulate, medial forebrain bundle, NAcc	Deficits in attention, memory, processing speed, executive function [40,45]	Some improvement in memory, attention, executive functioning up to 18 months [40]	Rare cases of hypomania with parameter settings [50]	Improvements often align with mood recovery rather than direct cognitive modulation [40]
Tourette’s syndrome (TS)	Thalamus, GPi, NAcc	Attention and executive deficits, especially in comorbid OCD/ADHD [26,42]	DBS may improve inhibitory control and tic-related cognitive load	Some cases of cognitive slowing reported, but not consistent	Difficult to isolate DBS effect vs. baseline comorbidities (OCD, ADHD)
Anorexia nervosa	Subcallosal cingulate	Rigid thinking, attentional bias, cognitive inflexibility [54,55,56]	Improved affect regulation and flexibility with symptom improvement [54,55,56]	Limited; some non-responders show no benefit	Cognitive improvement likely secondary to weight/mood recovery [54,55,56]
Addiction (heroin, cocaine)	NAcc	Impaired decision-making, reward processing [41,42]	Improved cognitive control via craving reduction [41,42]	Evidence preliminary; no consistent adverse effects reported	Differentiation requires larger controlled studies [41,42]

Abbreviations: STN—subthalamic nucleus; GPi—globus pallidus internus; VIM—ventral intermediate nucleus of the thalamus; NAcc—nucleus accumbens; ANT—anterior nucleus of the thalamus; CM—centromedian nucleus of the thalamus; NBM—nucleus basalis of Meynert; DBS—deep brain stimulation; OCD—obsessive–compulsive disorder; TRD—treatment-resistant depression; TS—Tourette’s syndrome; AD—Alzheimer’s disease; QoL—quality of life.

## Data Availability

No new data was created or analyzed in this study. Data sharing is not applicable to this article.
